# In situ quantification of poly(3-hydroxybutyrate) and biomass in *Cupriavidus necator* by a fluorescence spectroscopic assay

**DOI:** 10.1007/s00253-021-11670-8

**Published:** 2022-01-11

**Authors:** Alexander Kettner, Matthias Noll, Carola Griehl

**Affiliations:** 1 Competence Center Algal Biotechnology, Anhalt University of Applied Sciences, Bernburger Strasse 55, 06366 Koethen, Germany; 2 Institute of Bioanalysis, Coburg University of Applied Sciences and Arts, Friedrich-Streib-Str. 2, 96450 Coburg, Germany

**Keywords:** *Cupriavidus necator*, Polyhydroxyalkanoates, Fluorescence spectroscopy, BODIPY^493/503^, LipidGreen2, Nile red

## Abstract

**Abstract:**

Fluorescence spectroscopy offers a cheap, simple, and fast approach to monitor poly(3-hydroxybutyrate) (PHB) formation, a biodegradable polymer belonging to the biodegradable polyester class polyhydroxyalkanoates. In the present study, a fluorescence and side scatter-based spectroscopic setup was developed to monitor in situ biomass, and PHB formation of biotechnological applied *Cupriavidus necator* strain. To establish PHB quantification of *C. necator*, the dyes 2,2-difluoro-4,6,8,10,12-pentamethyl-3-aza-1-azonia-2-boranuidatricyclo[7.3.0.03,7]dodeca-1(12),4,6,8,10-pentaene (BODIPY^493/503^), ethyl 5-methoxy-1,2-bis(3-methylbut-2-enyl)-3-oxoindole-2-carboxylate (LipidGreen2), and 9-(diethylamino)benzo[a]phenoxazin-5-one (Nile red) were compared with each other. Fluorescence staining efficacy was obtained through 3D-excitation-emission matrix and design of experiments. The coefficients of determination were ≥ 0.98 for all three dyes and linear to the high-pressure liquid chromatography obtained PHB content, and the side scatter to the biomass concentration. The fluorescence correlation models were further improved by the incorporation of the biomass-related side scatter. Afterward, the resulting regression fluorescence models were successfully applied to nitrogen-deficit, phosphor-deficit, and NaCl-stressed *C. necator* cultures. The highest transferability of the regression models was shown by using LipidGreen2. The novel approach opens a tailor-made way for a fast and simultaneous detection of the crucial biotechnological parameters biomass and PHB content during fermentation.

**Key points:**

• *Intracellular quantification of PHB and biomass using fluorescence spectroscopy.*

• *Optimizing fluorescence staining conditions and 3D-excitation-emission matrix.*

• *PHB was best obtained by LipidGreen2, followed by BODIPDY*^*493/503*^* and Nile red.*

**Graphical abstract:**

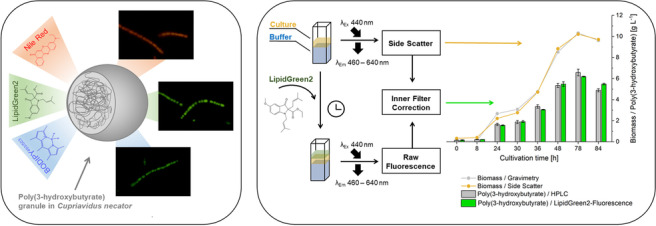

**Supplementary Information:**

The online version contains supplementary material available at 10.1007/s00253-021-11670-8.

## Introduction

Over the last decades, fluorescence spectroscopy has become an essential analytical tool in biochemical and biotechnological studies due to the technique’s high specificity and sensitivity. Moreover, fluorescence-based monitoring is non-destructive, rapid, and requires no extensive sample preparation before analysis (Hudson et al. 2007). All metabolites capable of fluorescence can be analyzed, making it suitable to monitor physiological states and microorganisms’ compounds during the fermentation. These include the determination of primary fluorophores like carotenoids, proteins, or NADPH (Schneckenburger et al. 1985; Simis et al. 2012). To analyze non-fluorescent substances, fluorescent dyes have to be linked to the target metabolites (Fam et al. 2018). This strategy is needed when stress-related metabolites, like lipid droplets formation or polyhydroxyalkanoate (PHA) accumulation, are addressed (Garay et al. 2014). The fully biodegradable biopolymer class PHA is becoming increasingly important as a sustainable counterpart to environmentally unfriendly conventional plastics (Koller et al. 2017; Dietrich et al. 2017; Raza et al. 2018; Anjum et al. 2016). PHA, as natural polyesters, is produced by prokaryotes as carbon and energy storage, like *Cupriavidus necator* (Chakraborty et al. 2012; Azubuike et al. 2020). The PHA biosynthesis is primarily induced by a macro-nutrient deficiency, including nitrogen or phosphorus, and a simultaneous oversupply of carbon sources. The polyesters are deposited intracellularly in the form of lipophilic organelle-like granules (Bresan et al. 2016). Depending on the microorganism and its cultivation conditions, homo- or co-polyesters with a wide variety of 3-hydroxyalkanoic acids and correspondingly variable mechanical properties can be found (Tan et al. 2014).

The best-studied representative is poly(3-hydroxybutyrate) (PHB). PHB and other PHA representatives are already industrially manufactured (McAdam et al. 2020). However, the production and application lag behind the potential (Zheng et al. 2020). The main reasons are the high production costs related to fermentation and downstream processing (Atlić et al. 2011; Johnson et al. 2009; Oliveira et al. 2007; Koller and Braunegg 2018). A fluorescence-based methodology to detect PHB intracellular can significantly reduce the costs by determining the optimal harvest time during the fermentation process as a fast and cheap on-line approach. To detect PHB with fluorescence, several dyes have shown to be suitable. The best-studied and most applied probe is the Nile blue oxazone Nile red (9-(diethylamino)-5H-benzo[a]phenoxazin-5-one; NR), which shows a bright yellow to red fluorescence signal emission (Greenspan and Fowler 1985; Gorenflo et al. 1999; Spiekermann et al. 1999). NR was described as an environmental-sensitive, solvatochromic probe, indicated by a bathochromic emission shift and a reducing emission intensity when environmental polarity increases. Hence, the emission wavelength maximum reduces when dye molecules accumulate in lipophilic PHB-granules. Solvatochromic dyes are therefore favorable since a low background fluorescence occurs during the detection of PHB. However, NR indicates several limitations, like non-specific staining of other cell components (membrane lipids) and dye concentration-related anomalies. These characteristics complicate a method’s reproducibility (Collot et al. 2018; Pick and Rachutin-Zalogin 2012).

Boron-dipyrromethene fluorescence dyes (BODIPY-dyes) are well-known for a better cell permeation and a higher affinity to lipophilic substances than NR (Rumin et al. 2015; Cirulis et al. 2012). These intrinsically high lipophilic organoboron compound class show a remarkably high quantum yield, and a small stokes shift, and, unlike Nile red, emit in the green spectral range (Koreivienė 2020). Recently, the non-polar BODIPY-derivate BODIPY^493/503^ (4,4-Difluoro-1,3,5,7,8-pentamethyl-4-bora-3a,4a-diaza-s-indacene, BY) was described for PHB detection (Zhu et al. 2011; Brown et al. 2020).

However, BODIPY dyes demonstrate a high background fluorescence in a polar environment (Zhu et al. 2011). The respective dyes’ limitations promoted the development of new probes in all spectral areas (Klymchenko et al. 2012).

A novel green fluorescent N-dimethylallylated 3-hydroxyindole-based molecule LipidGreen2 (Ethyl 5-methoxy-1,2-bis(3-methylbut-2-enyl)-3-oxoindole-2-carboxylate; LG2) was recently developed from LipidGreen1 (Chun et al. 2013). Both probes had been successfully applied for lipid droplet staining in cell lines as well as PHB screening and monitoring approaches in *Escherichia coli* and *C. necator* (Choi et al. 2015; Lee et al. 2011; Kettner and Griehl 2020). LG2 demonstrated a higher hydrophobicity, sensitivity, and fluorescence efficiency for lipid droplets with a low background fluorescence compared to BY (Chun et al. 2013). Nevertheless, comparative studies between BY and LG2 during PHB detection staining have not yet been described.

Regardless of the chosen probe, the main limitation of dye-based PHB quantification displays the methodology’s reproducibility (Resch-Genger et al. 2005). This is based on the staining process complexity, which can be classified into biotic and abiotic factors. While abiotic factors such as probe concentration and diffusion (incubation time) can be adjusted and controlled, biotic factors can change during the cultivation or in comparison to different cultures. Therefore, the impact of the factors cannot be underrated or neglected. During the fermentation process of *C. necator*, following biological states can occur: (i) cell concentration > product concentration (growth phase), (ii) cell concentration ~ product concentration (stationary phase), and (iii) cell concentration < product concentration (death phase). However, the biomass concentration directly affects the fluorescence and can negatively affect the PHB emission signal. These so-called inner filter effects (IFE) scatter both excitation and emission and increases as biomass concentration increases (Panigrahi and Mishra 2019). To overcome these limitations, a constant optical density (OD) of the samples can be applied. However, an OD adjustment also increases the potential for errors, reduces reproducibility, and represents a further manual step (Larsson et al. 2007).

Hence, all mentioned factors require methodological fine-tuning to establish an optimized fluorescence staining procedure and guarantee a robust transferability and reproducibility.

This work aimed to develop a fluorescence spectroscopic assay for fast and reproducible monitoring of bacterial PHB and biomass content during the cultivation of *C. necator*. For this purpose, the fluorescent probes BY, LG2, and NR were compared, and the effect on the staining efficiency was analyzed, adjusted, and IFE corrected. Afterward, the developed method was applied to different PHB formation strategies to investigate the reproducibility and transferability of the overall developed methodology.

## Material and methods

### Chemicals

If not stated else, chemicals were analytical grade and obtained either from Carl Roth (Karlsruhe, Germany), Merck (Darmstadt, Germany), or Avantor (Hannover, Germany). BY and NR were purchased from Thermo Fisher Scientific (Waltham, USA) and LG2 from Merck (Darmstadt, Germany).

### Strains and cultivations

Synthesis of PHB was conducted by *C. necator* DSM 545, which was obtained from the German Collection of Microorganisms and Cell Cultures (DSMZ, Braunschweig, Germany). The cultivation was performed in three stages, each with a defined media composition. First, a starter culture of *C. necator* was grown for 48 h and mixed at 180 rpm at 30 °C (Table 1). In a second step, the culture was transferred into the seed culture medium with 10% inoculum and cultivated for 48 h, slightly modified, as described previously (Ryu et al. 1997). Finally, the formation of PHB was induced by batch cultivation at 30 °C and 250 rpm in 2 L aerated bioreactor under N-deficit conditions. P-deficit and NaCl stress conditions were further used in the subsequent experiments. The pH of all cultures was initially set to 7.2 ± 0.2 by adding 2 M NaOH.

**Table 1:** Composition of the cultivation media. All media included trace element solution that consisted of 10 g L^−1^ FeSO_4_(7H_2_O), 2.25 g L^−1^ ZnSO_4_(7H_2_O), 1 g L^−1^ CuSO_4_(5H_2_O), 0.5 g L^−1^ MnSO_4_(5H_2_O), 2 g L^−1^ CaCl_2_(2H_2_O), 0.23 g L^−1^ Na_2_B_4_O_7_(7H_2_O), 0.1 g L^−1^ (NH_4_)_6_Mo_7_O_24_, and 10 mL L^−1^ 35% HCl as reported earlier (Kimm et al. 1993)

Media component	Start culture	Seed culture	N-deficit	P-deficit	NaCl-stress
Peptone	5.0 g L^−1^				
Meat extract	3.0 g L^−1^				
Na_2_HPO_4_ (12H_2_O)		9.0 g L^−1^	9.0 g L^−1^	-	9.0 g L^−1^
KH_2_PO_4_		1.5 g L^−1^	1.5 g L^−1^	0.5 g L^−1^	1.5 g L^−1^
(NH_4_)_2_SO_4_		1.0 g L^−1^	0.5 g L^−1^	4.0 g L^−1^	0.5 g L^−1^
MgSO_4_ (7H_2_O)		0.2 g L^−1^	1.2 g L^−1^	1.2 g L^−1^	1.2 g L^−1^
Citric acid		-	1.7 g L^−1^	1.7 g L^−1^	1.7 g L^−1^
NaCl		-	-	-	2.5 g L^−1^
Trace element solution		1 mL	10 mL	10 mL	10 mL
Glucose/fructose		5/5 g L^−1^	10/10 g L^−1^	10/10 g L^−1^	10/10 g L^−1^

### Biomass and PHB-determination

Cultivation broth was determined gravimetrically. Briefly, 3 to 9 mL of biomass was stepwise centrifuged (Thermo Scientific, Waltham, USA) at 5000x*g* for 10 min in micro reaction tubes (VWR, Darmstadt, Germany), and the remaining pellet was washed twice with distilled water, frozen, lyophilized (Christ Martin, Osterode, Germany), and weighed. Biomass concentration was calculated from the ratio of dried biomass to cell culture volume. PHB was quantified by high-performance liquid chromatography (HPLC) as previously described (Karr et al. 1983) using the following modifications: 1 mL of 75% sulfuric acid was added to the retained dried biomass and heated at 95 °C for 60 min. Dehydrated biomass was diluted with distilled water before measurement. Solid PHB standard was treated alike and used for calibration. As the PHB hydrolysis product, crotonic acid (cis and trans isomer, Fig. S1) was quantified with a Merck-Hitachi HPLC equipped with a UV detector at 214 nm. Chromatographic separation was isocratically performed with ABOA SugarSep column (AppliChrom, Oranienburg, Germany) at a flow rate of 0.8 mL min^−1^, 60 °C, 50 bar, and 0.007 N H_2_SO_4_ as mobile phase.

### Fluorescence device and sampling set up

Optical fluorescence measurements were performed with a Perkin Elmer LS45 (Perkin Elmer, Waltham, USA) using standard 1.0 cm path length cuvettes (Brand, Wertheim, Germany). For all experiments, excitation and emission slits were set to 10 nm at a gain of 650 V. The PHB and biomass measurements were performed in two successive steps using the identical device setup/spectral monochromator constellation. First, the side scatter (SSC) was measured, and after the fluorescent probe was added and the incubation time passed, the fluorescence emission was recorded. Optimal excitation and emission and solvatochromic behavior of the dyes were studied with 3D-excitation-emission-matrix (3DEEM). Therefore, concentrations of 1.0 µg mL^−1^ (NR, LG2) and 0.1 µg mL^−1^ (BY), 5% DMSO, and 30 min incubation were applied, and the spectral range between 300 and 800 nm was scanned. Ideal excitation wavelengths were selected from the received 3DEEM and emission was recorded over 150 nm starting 20 nm after excitation.

To study the staining conditions and impact of abiotic and biotic parameters, design of experiments (DoE) was used. The cell suspension was mixed with buffer, fluorescence dye, DMSO, and isopropanol to archive the conditions of the plan (Table 2). The fluorescent probes were diluted in DMSO to obtain a 20 µg mL^−1^ (BY) or 200 µg mL^−1^ (LG2, NR) stock solution. The buffers phosphate-buffered saline (PBS I) containing 2.34 mg L^−1^ NaCl, 0.04 mg L^−1^ KCl, 0.07 mg L^−1^ Na_2_HPO_4_, and 0.01 mg L^−1^ KH_2_PO_4_ and PBS II consisting of PBS I, 0.03 mg L^−1^ EDTA, and 0.08 mg L^−1^ Tris at a pH of 7.5 were tested. Incubation was carried out under light exclusion at room temperature for 1, 2, 5, and 10 min. DoE was planned and conducted with Visual-XSel 15 software (CRGRAPH, Starnberg, Germany) and evaluated by partial least square and the program’s parameter optimization tool to obtain the final staining parameters for subsequent experiments.

**Table 2:**
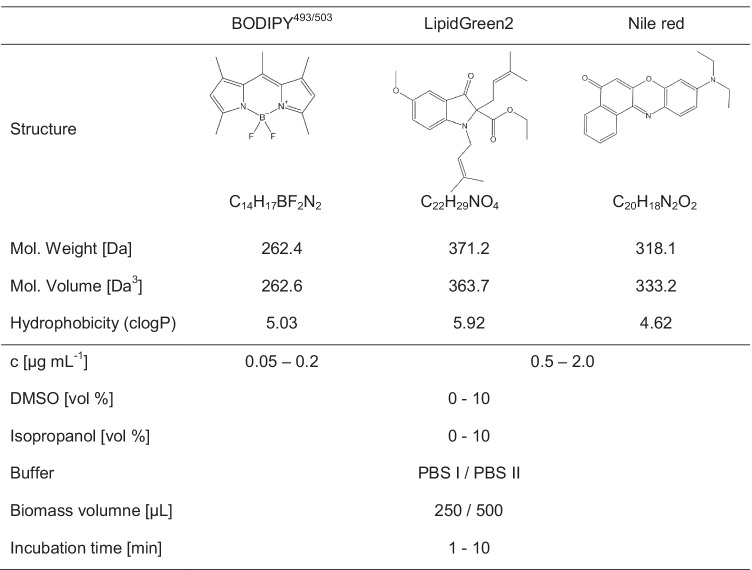
Molecular properties of the used fluorescence dyes in this study and DoE parameter range for staining optimization. Chemical information were calculated with ChemDraw ultra. Abbreviations: c, fluorescence dye concentration; *DMSO*, dimethyl sulfoxide. Buffer types: phosphate buffer saline (PBS I) and phosphate buffer saline including 0.03 mg L^−^ EDTA and 0.08 mg L^−1^ Tris (PBS II), both at pH 7.5

### Fluorescence-microscopy

Epi-fluorescence microscopic analysis was performed with an Olympus BX41 (Olympus, Tokyo, Japan) to visualize intracellularly accumulated PHB granules. Excitation filters of 400–440 nm for BY and 460–490 nm for LG2 and NR were applied. According to the manufacturers’ instructions, images were captured with an Olympus XC50 camera and analyzed with the cellSens standard software (Olympus).

### Data processing

To reduce the impact of fluorescence setup-related IFE and to increase the linear relationship between PHB content and fluorescence signal, the raw data were corrected with and without incorporating the SSC using the following mathematical equations: square root of raw fluorescence (S_RF), logarithm of raw fluorescence (ln_RF), harmonic mean (HM), arithmetic mean (AM), square root of arithmetic mean (S_AM), product (PR), square root of product (S_PR), quotient (QU), square root of quotient (S_QU), difference (DI), and square root of difference (S_DI). Equations are given in supplementary information (Tab. S1). Data correlation, plotting, and artwork were performed with Origin 2020 Pro (OriginLab, Northampton, USA).

## Results

### Fluorescent dye characterization and staining protocol

**Fig. 1:**
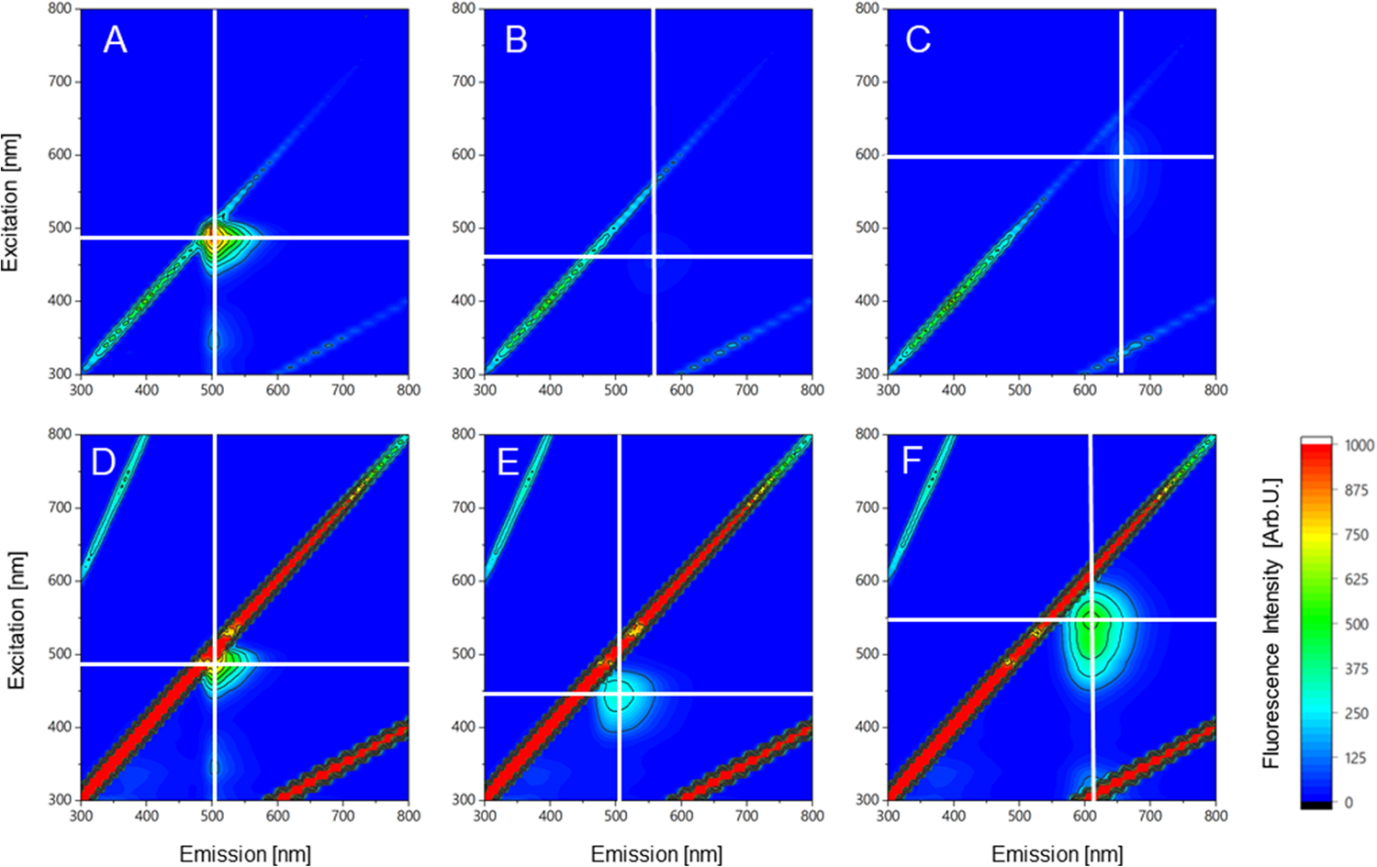
3D-excitation-emission matrix of BODIPY^493/503^ **(A, D)**, LipidGreen2 **(B, E)**, and Nile red **(C, F)** in phosphate buffer saline as polar (first row) and in PHB stored *C. necator* as nonpolar environment (second row). Fluorescence intensities are shown as false colors. The white cross indicates the highest fluorescence emission

3DEEM were recorded using PBS buffer as a polar and PHB-enriched *C. necator* as a lipophilic environment to investigate solvatochromism and optimal excitation wavelength. Ideal excitation for PHB quantification was observed at 440–460 nm (BY), 420–450 nm (LG2), and 500–550 nm (NR) (Fig. 1). Both LG2 and NR indicated a solvatochromic shift of approximately 50 nm, resulting in a low background fluorescence during PHB detection. BY indicated no environmentally sensitive solvatochromic behavior. The emission region and intensity were contrastable in the polar and nonpolar environments. BY indicated a very sharp and intensive emission with a small stokes-shift, based on a high quantum yield. LG2’s and NR’s 3D-peak areas were more extensive, ensuring higher measurement flexibility. LG2 displayed the lowest emission intensities of all tested dyes. To guarantee no crosstalk between the excitation and emission, wavelengths of 450 nm (BY), 440 nm (LG2), and 525 nm (NR) were chosen as excitation wavelengths for subsequent experiments.

**Fig. 2:**
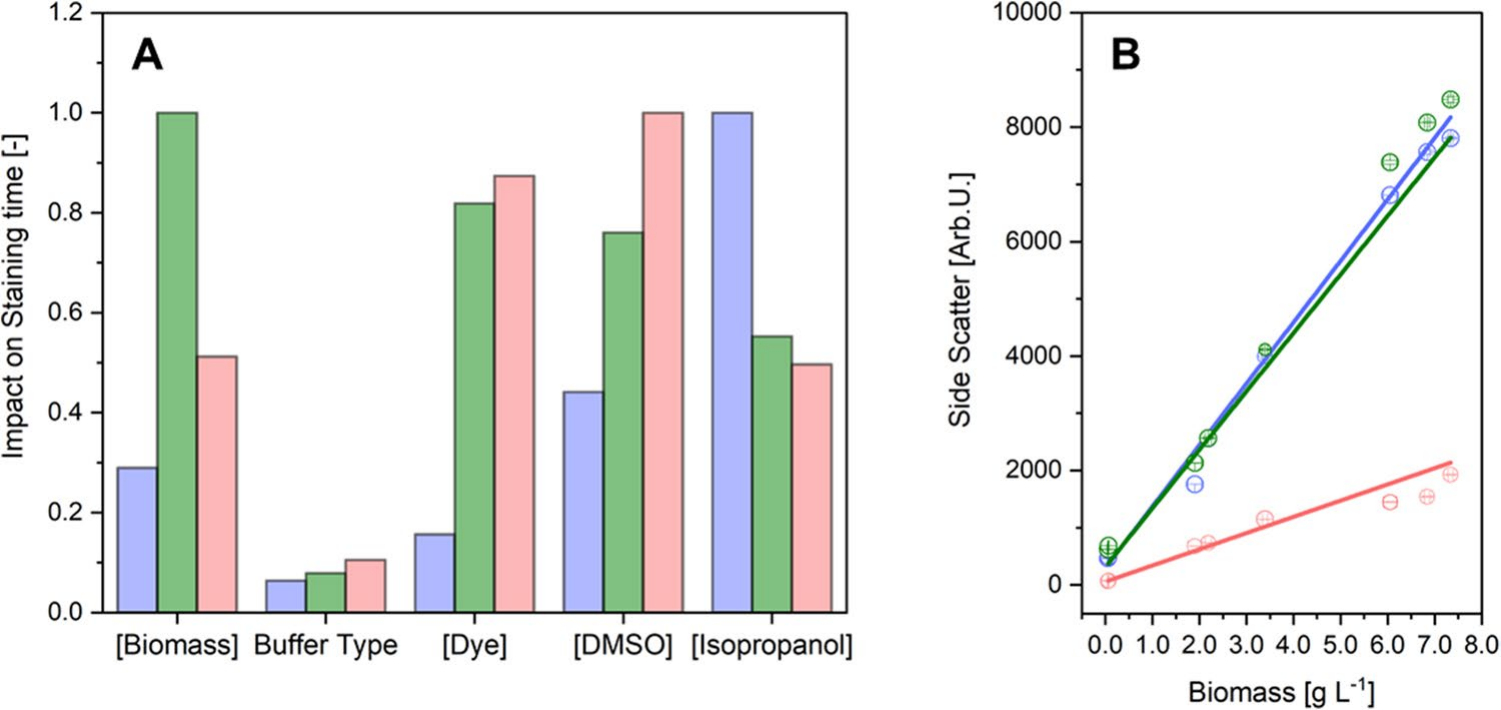
Normalized parameter’s impact on staining time depending on the tested fluorescence dye **(A)**. Impact was shown for the tested parameter ranges as summarized in Table 2. Buffer types: phosphate buffer saline and phosphate buffer saline, including 0.03 mg L^−1^ EDTA and 0.08 mg L^−1^ Tris, both at pH 7.5. DMSO, dimethyl sulfoxide. Linear regression of side scatter to biomass of N-depleted *C. necator* **(B)**. Dyes: BODIPY^493/503^ (blue, *R*^2^ = 0.9977), LipidGreen2 (green, *R*^2^ = 0.9841), Nile red (red, *R*^2^ = 0.9868). Error bars represent the standard derivation of 3 independent replicates

The DoE-based staining experiment revealed that the magnitude of each influencing factor was dependent on the respective fluorescent dye. For BY, the carrier solvents isopropanol demonstrated the highest impact (1.0) on the staining process (Fig. 2A). DMSO (0.44) and the biomass (0.29) revealed a minor effect; the buffer type (0.06) and the dye concentration (0.16) were deficient and, therefore, proved a subordinated role on the staining process. With LG2 and NR, more factors showed a superior effect compared to BY. All tested parameters exhibited an influence of more than 0.5 on LG2 and NR, while BY was primarily influenced by isopropanol. The cell concentration (1.0) affected the LG2-based PHB staining, followed by the dye and DMSO concentration (0.82; 0.76). For NR, the DMSO concentration (1.0) and the dye concentration (0.87) demonstrated the highest magnitudes of impact on the staining process. Compared to BY, isopropanol displayed levels of 0.55 and 0.50 lower impacts for LG2 and NR, respectively. Based on these findings, each dye’s appropriate staining conditions were determined using the software’s optimization tool (Tab. S2). Subsequent experiments were carried out with the obtained staining conditions at 5 min incubation (Table 3). The obtained staining protocols were also examined for bioimaging. The PHB granules could be clearly visualized with all three fluorescent dyes (Fig. S2).

**Table 3:** Best staining conditions after 5 min incubation based on DoE of Table 2 and Visual Xsel parameter optimization tool. Abbreviations: c, fluorescence dye concentration; DMSO, Dimethyl sulfoxide; Isoprop, Isopropanol; Buffer types: phosphate buffer saline (PBS I) and phosphate buffer saline, including 0.03 mg L^−1^ EDTA and 0.08 mg L^−1^ Tris (PBS II), both at pH 7.5

Dye	C [µg mL^−1^]	Buffer type	Biomass [µL]	DMSO [%]	Isopropanol [%]
BODIPY^493/503^	0.2	PBS II	500	9	10
LipidGreen2	1.0	PBS II	500	10	0
Nile red	1.0	PBS II	500	10	0

### Fluorescence sensor setup for N-depleted culture

The resulting protocol and spectral fluorescence parameters were applied to an N-depleted *C. necator* culture (Fig. S3). The SSC signal showed a strong linear relationship to the gravimetrically determined cell concentration (Fig. 2B). Therefore, SSC values were proportional to the biomass. However, the slopes of SSC signals slightly differed for BY (1073.4) and LG2 (1022.2) and was much lower for NR (283.3). The equations resulting from the regression models were afterward used to determine the biomass concentration in subsequent experiments.

**Fig. 3:**
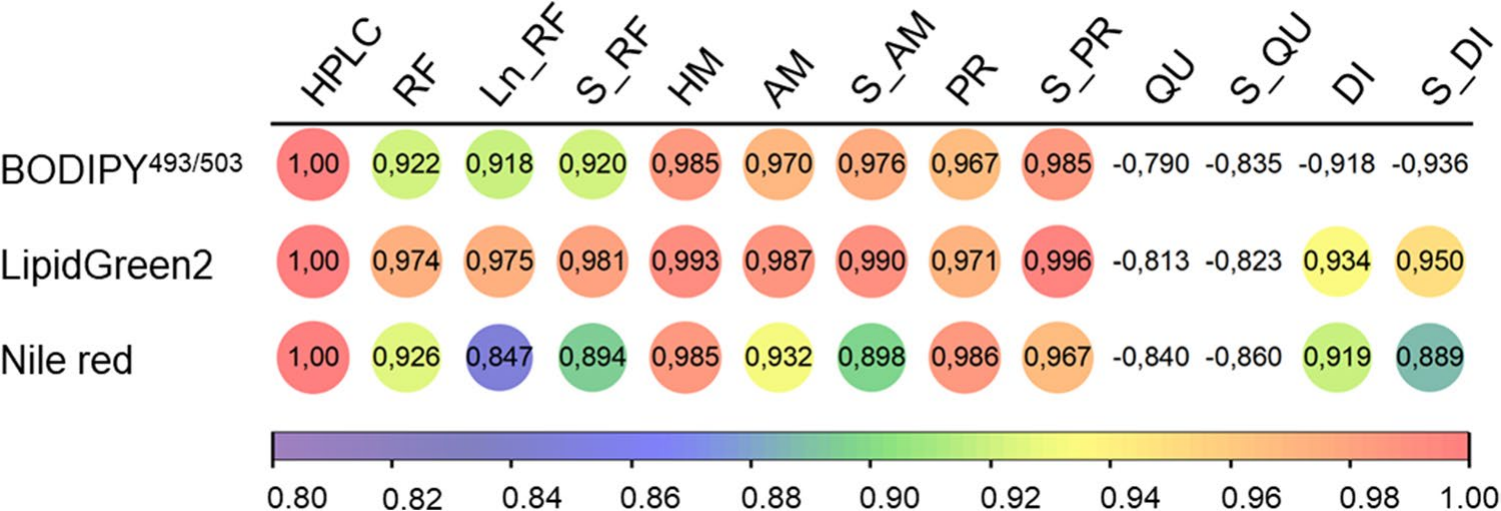
Correlation of HPLC measured PHB contents to the fluorescence calculated values using different inner filter corrections. For better visualization, values are false-colored. Legend: HPLC, PHB content obtained of HPLC measurements; RF, raw fluorescence; Ln_RF, logarithm of raw fluorescence; S_RF, square root of raw fluorescence; HM, harmonic mean of side scatter and raw fluorescence; AM, arithmetic mean of side scatter and raw fluorescence; S_AM, square root of the arithmetic mean; PR, product of side scatter and raw fluorescence; S_PR, square root of product; QU, quotient of raw fluorescence to side scatter; S_QU, square root of quotient; DI, difference of raw fluorescence to side scatter; S_DI, square root of difference of raw fluorescence to side scatter

Linear relationships between PHB-dye-complex’s raw fluorescence (RF) and the HPLC-measured PHB content could be achieved after 5 min of incubation for all tested dyes. Thereby, the probe LG2 yielded 0.974 in the highest correlation, while BY (0.922) and NR (0.926) displayed slightly lower correlations (Fig. 3).

SSC was used to correct the IFE-related impact to enhance the linear correlation between the RF emission and the PHB content. BY demonstrated the highest relationship to analytically determined PHB when the harmonic mean (0.985) and the square root of the product of RF and SSC (0.920) were applied. In comparison, the difference of the RF value to the SSC value or the square root of this difference did not indicate any correlation for BY fluorescence.

The RF exhibited for LG2-stained cells a good agreement with the analytically determined PHB concentration (0.974). The correlation could be increased to 0.993 and 0.996 when applying the harmonic mean and the square root of this difference, respectively. Besides applying the quotient of RF to SSC or the square root of this quotient, all tested correction models resulted in linear correlations higher than 0.97 for LG2. The highest correlation values for NR staining were achieved when the harmonic mean, the product, and the square root of the product of RF and SSC were employed. Since the harmonic mean and the square root of the product as fluorescence signal correction methods displayed the highest correlations for all probes, these methods were deployed to correct IFE in subsequent experiments.

### Application of fluorescence sensor to different cultures

**Fig. 4:**
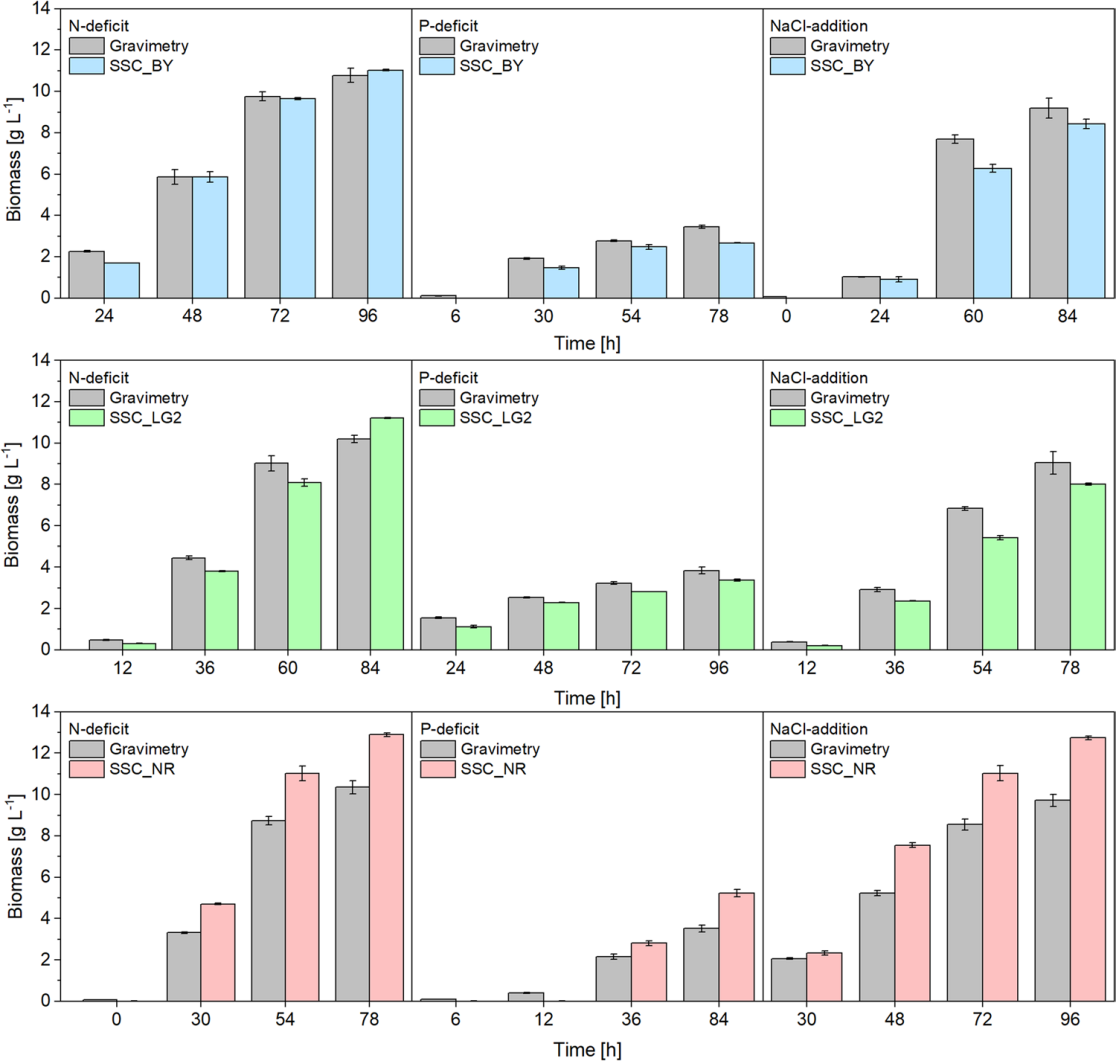
Comparison of side scatter obtained biomass contents in different depletion cultures compared to gravimetric analysis (gray bars) using the spectral settings of BODIPY^493/503^ (blue), LipidGreen2 (green), and Nile red (red). Error bars represent the standard derivation of 3 independent replicates

To examine the transferability and reproducibility of the developed methodology, the developed fluorescence-based measurement was deployed to N-depleted, P-depleted, and NaCl-stressed *C. necator* cultures. The previously created regression models were used for biomass and PHB content monitoring. The employed linear regression models showed a high-level accuracy of the BY and LG2 SSC-based biomass determination compared to the gravimetrically obtained values (Fig. 4). The highest agreement between the gravimetrically measured biomass and SSC-based measurement was achieved with the N-depleted culture. BY and LG2 revealed an agreement of 94% to this culture, while for NR, 87% was just reached. The general agreement of the SSC biomass sensor to the gravimetrically obtained results overall tested cultures was 85% for BY, 84% for LG2, and 76% for NR.

**Fig. 5:**
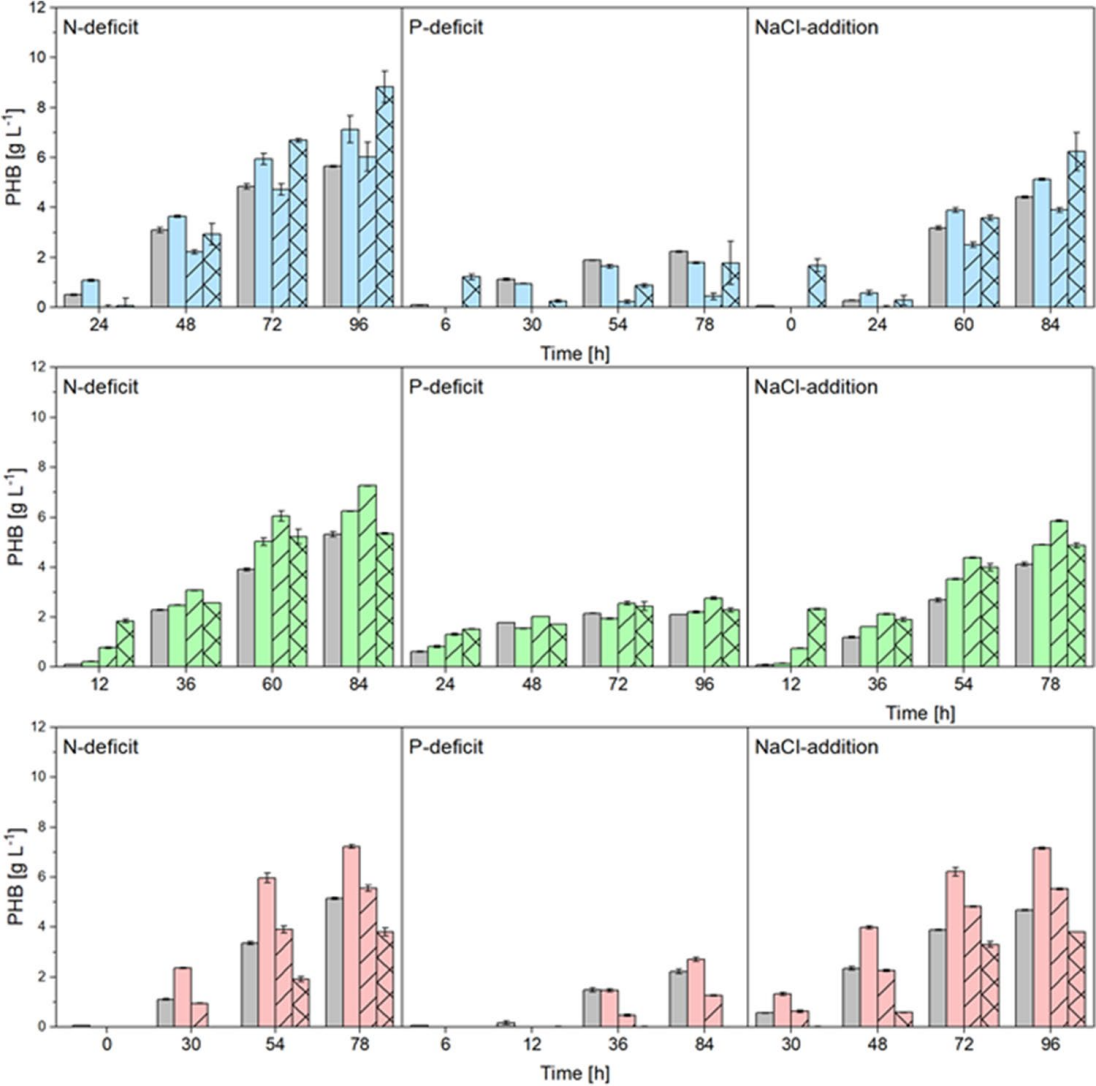
Comparison of calculated PHB contents using BODIPY^493/503^ (blue), LipidGreen2 (green), and Nile red (red) for different cultivation conditions of *C. necator* (N-deficit, P-deficit, and NaCl-addition). The raw data were corrected with the harmonic mean of side scatter and raw fluorescence (solid bars) and the square root of the product of both (dashed bars), compared to raw fluorescence values (crossed bars) and the analytical reference (HPLC, gray bars). Error bars represent the standard derivation of 3 independent replicates

The regression models of the harmonic mean and the square root of the product of SSC and RF were applied, and the resulting values were compared to analytically obtained PHB concentration (Fig. S4). LG2 demonstrated contrastable calculated PHB levels in all three independent replicates cultures, whereby the harmonic mean represented a suitable IFE correction method (Fig. 5). However, even when the RF was used directly, an appropriate relationship was achieved, despite the lower coefficient of determination of the previously obtained calibration culture. A constant detection of PHB indicated a good transferability with LG2 to all deficient cultures.

## Discussion

In the first step, solvatochromism and optimal excitation wavelength were studied through 3DEEM (Fig. 1). The resulting and applied wavelengths of 450 nm (BY), 440 nm (LG2), and 525 nm (NR) can be confirmed through comparative approaches, whereby excitation for NR ranged from 488 to 540 nm, while 450 nm and 440 nm were reported for BY and LG2, respectively (Sitepu et al. 2012; Spiekermann et al. 1999; Wu et al. 2003; Govender et al. 2012). 3DEEM approved polarity-sensitive solvatochromism of NR’s and LG2’s due to intrinsic biophysical push–pull structural properties (Klymchenko 2017). In contrast, BY did not demonstrate solvatochromism. Environmental sensitive properties are privileged for PHB quantification since a differentiation to the molecules that did not bind to the hydrophobic PHB granule can be obtained (Danylchuk et al. 2019; Wu et al. 2003). BY indicated a high background fluorescence, which makes this dye less useable for standard cuvette-based fluorescence measurements. Anyway, BY dyes were successfully applied in several publications, demonstrating a good dye performance for lipophilic target quantification on single cell level (Brennan et al. 2012; Beatriz 2013; Karmann et al. 2016). The BY dye concentration, buffer type, and biomass concentration was subordinate in the staining process (Fig. 2A). The resulting short incubation time and high emission intensities can be explained by the low molecular volume and high hydrophobicity index, which may enhance cell membrane penetration.

Due to the significantly higher molecular weight of LG2 and NR, the biomass and the dye’s concentration play a more critical role in the staining procedure. Especially for LG2 staining, which has the highest molecular weight of all tested dyes, it is decidedly impacted by the biomass concentration (Fig. 2A).

The biomass determination and the fluorescence-based quantification of PHB were successful with all three dye’s excitation and emission ranges (Fig. 2B). It turned out that biomass determination is preferable to carry out at a shorter wavelength since the equations’ slope was higher due to higher signal intensities than in the red spectrum. The SSC-based measurements of biomass were, therefore, best with BY and LG2 parameter constellation. However, the low measured values for the NR range, that based on the low regression slope, promoted a higher derivation of 17%. The modest fluorescence values in the red spectrum can be explained by the poor sensitivity of the photomultiplier detector in the red region, which is about 50% lower when compared to the blue and green region (Perkin Elmer 2007). The applied linear regression models obtained from the N-depleted *C. necator* culture were applicable on the other depleted and stressed cultures (Fig. 4), indicating the universal applicability of the side scatters as a biomass sensor for *C. necator*. The use of the cell side scatter was also applied in flow cytometry, where the side scatter is a dimension of the cell granularity, quantity, and size (Vees et al. 2020).

A direct relationship between the RF and the PHB concentration is challenging to achieve (Fig. 3). To improve the linear relationship, different correction methods were applied and compared to the analytically determined values. The here applied correction methods can be divided into two categories. In the first, only the probe descends RF value is used, and in the second both, the RF and SSC. It can be stated that the second category, which applied the inclusion of SSC values to incorporate the inner filter effects, turned out in higher correlations for all tested dyes (Fig. 3). IFE are a known negative factor influencing the transferability and reproducibility in spectroscopy (Panigrahi and Mishra 2019). Several approaches have been found to be successful and consider particle scattering as measured by absorbance or Raman scattering (Goletz et al. 2011; Larsson et al. 2007). The method described here is advantageous since the same device and setup can be applied, which showcases a higher measurement accuracy and velocity. Thereby, the harmonic mean of side scatter value and RF, and the square root of the product of both had proven to be good fluorescence correction equations, resulting in high correlation coefficients and the re-applicability of the regression models (Fig. 5). The quality of regression models and the transferability to different depletion cultures depended more on the dye than on the selected IFE correction method. Nile red displayed the lowest transferability to the other depletion cultures as the RF and the SSC intensities are lower than those of BY or LG2, leading to higher derivations. Moreover, NR exhibits concentration-related anomalies and crystallizes at higher concentrations while quenching the fluorescence emission signal (Ray et al. 2019). Due to the high fluorescence quantum yield, BY can be a good choice for low concentrated targets on single-cell level, like flow cytometry, while Nile red shows excellent eligibility in fluorescence image analysis and polarity differentiation of intracellular lipids (Diaz et al. 2008; Maes et al. 2017).

LG2 revealed excellent transferability. Moreover, due to the higher hydrophobicity index, LG2 stained PHB-granules selectively, while NR had a lower affinity reported, resulting in staining both storage and membrane lipids (Pick and Rachutin-Zalogin 2012). In summary, it can be stated that the choice of dye had the most significant impact on the transferability. LG2 displayed favorable properties for the developed fluorescence spectroscopic setup. LG2 combined the positive characteristics of BY (blue/green spectral range) and NR (solvatochromism) and further demonstrated high correlations to analytically measured PHB concentrations. This new and fast (less than 20 min) approach is automatable as at-line sensors. Further research will focus on the devolution of the methodology to other PHA representatives like poly(3-hydroxybutyrate-co-4-hydroxybutyrate) or poly(3-hydroxybutyrate-co-3-hydroxyvalerate), which demonstrate better material and processing properties for medical and industrial applications than pure PHB.

## Supplementary Information

Below is the link to the electronic supplementary material.

Supplementary file1 (PDF 533 KB)

## Data Availability

All data generated or analyzed during this study are included in this published article and its supplementary information.
