# Supplementary material for: In situ quantification of poly(3-hydroxybutyrate) and biomass in Cupriavidus necator by a fluorescence spectroscopic assay
Source: Appl Microbiol Biotechnol. 2022 Jan 11;106(2):635–45. doi: 10.1007/s00253-021-11670-8 (PMC8763931; doi:10.1007/s00253-021-11670-8)
Supplement: Supplementary file 1 — Supplementary file1 (PDF 533 KB) [file 253_2021_11670_MOESM1_ESM.pdf]

Journal of Applied Microbiology and Biotechnology

In situ quantification of poly (3-hydroxybutyrate) and biomass in *Cupriavidus necator* by a fluorescence spectroscopic assay

Alexander Kettner[1], Matthias Noll[2], Carola Griehl[1]*

[1]Competence Center Algal Biotechnology, Anhalt University of Applied Sciences, Bernburger Strasse 55, 06366 Koethen, Germany

[2]Institute of Bioanalysis, Coburg University of applied Sciences and Arts, Friedrich-Streib-Str. 2, 96450 Coburg, Germany

*Corresponding author, Email: carola.griehl@hs-anhalt.de, Tel.: +49 (0) 3496 – 67 25 26

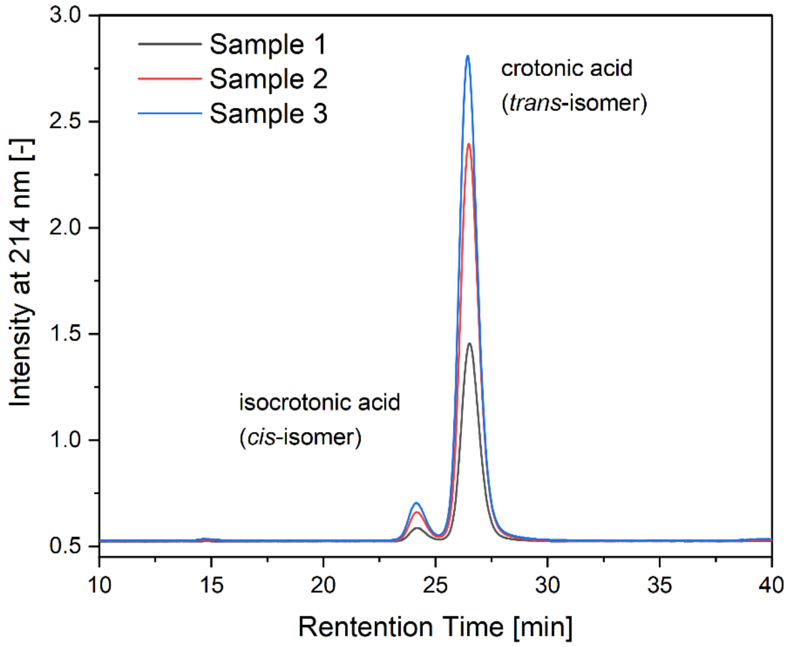

Figure S1 Chromatogram of isocrotonic and crotonic acid as the hydrolysis products of 2 mg mL^{-1} (Sample 1, black), 4 mg mL^{-1} (Sample 2, red), and 6 mg mL^{-1} (Sample 3, blue) poly(3-hydroxybutyrate), detected at 214 nm.

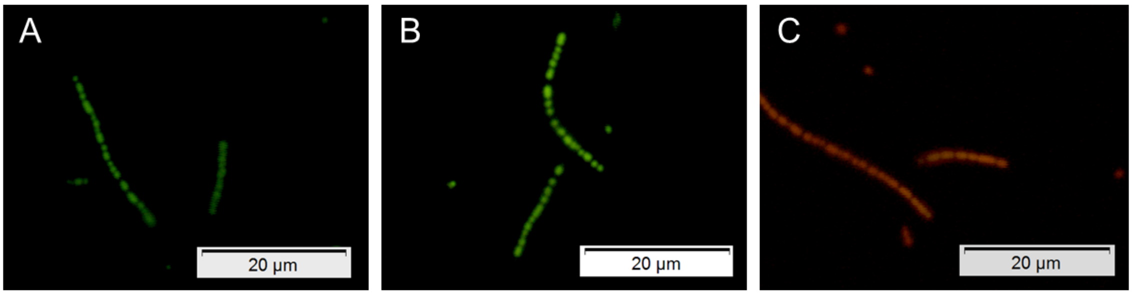

Figure S2 Fluorescent bioimaging of dye-stained PHB granules in *C. necator* using **(A)** BODIPY$^{493/503}$, **(B)** LipidGreen2, and **(C)** Nile red.

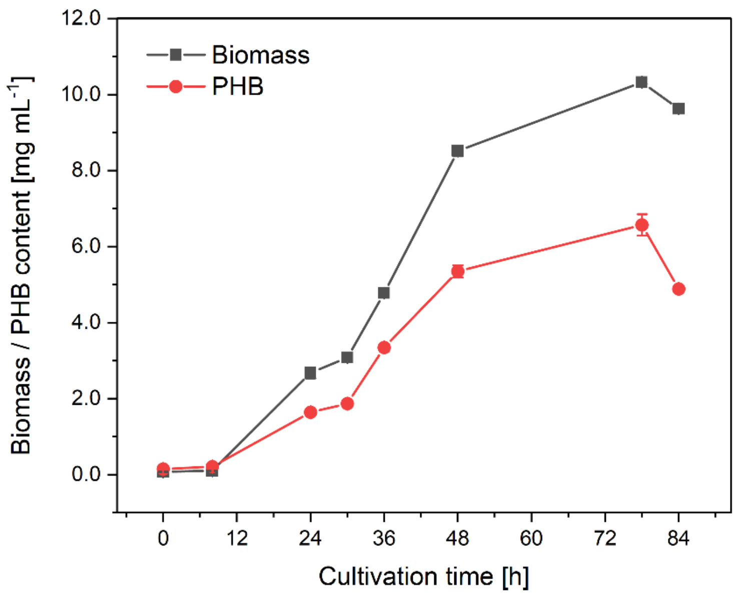

Figure S3 Biomass and PHB formation of N-deficit *C. necator* culture. See further details in main manuscript. Error bars represent the standard derivation of 3 independent replicates.

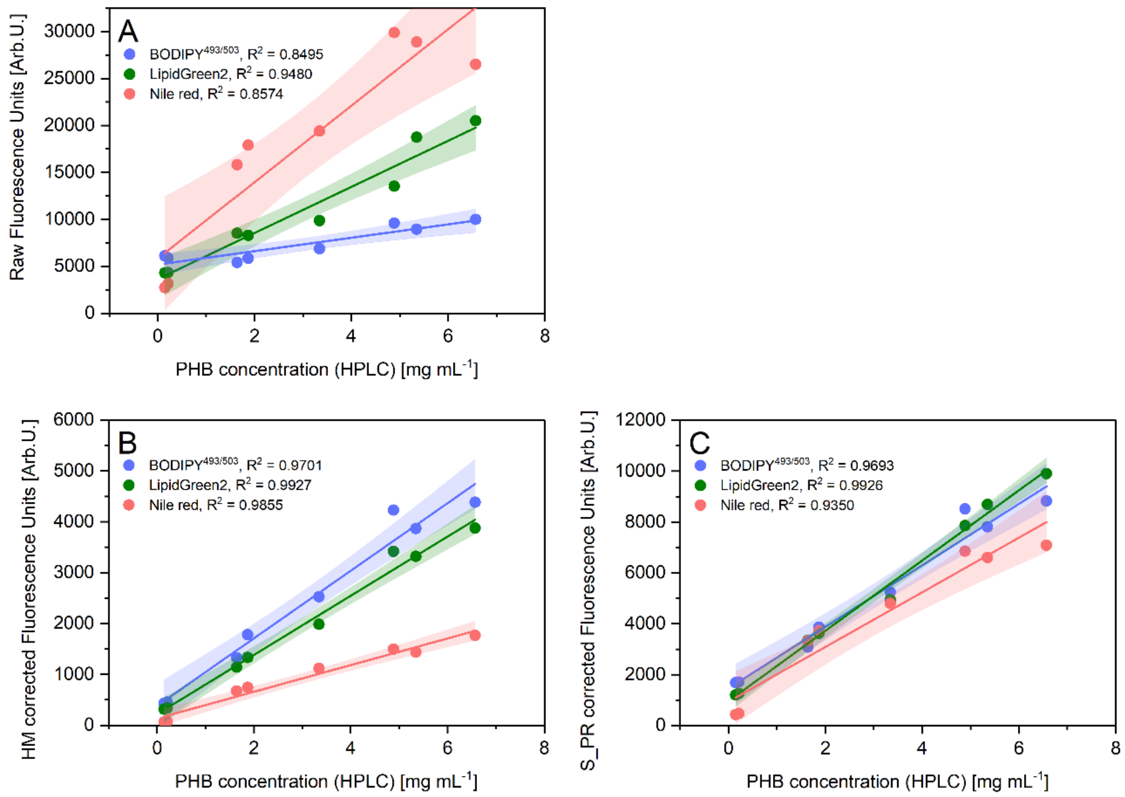

Figure S4 Linear regression models of fluorescence to HPLC measured Poly(3-hydroxybutyrate) concentrations. Data were obtained with BODIPY$^{493/503}$ (blue) LipidGreen2 (green), and Nile red (red) using the raw fluorescence **(A)**, the harmonic mean of raw fluorescence and fluorescence side scatter (HM) **(B),** and the square root of the product (S_PR) of both **(C).** The mean of three measurements is denoted, and the 95 % confidence interval is displayed in shaded color.

Table S1 Applied mathematical equations for correction of inner filter effects. RF: raw fluorescence signal; SSC: side scatter signal.

Name	Abbreviation	Equation
Square root of RF	S_RF	$S\_RF = \sqrt{RF}$
Logarithm of RF	Ln_RF	$\ln\_RF = lnRF$
Harmonic mean of RF and SSC	HM	$HM = (RF \cdot SSC)(RF + SSC)^{-1}$
Arithmetic Mean of RF and SSC	AM	$AM = \frac{1}{2}(RF + SSC)$
Square root of Arithmetic Mean of RF and SSC	S_AM	$S\_AM = \sqrt{\frac{1}{2}(RF + SSC)}$
Product of RF and SSC	PR	$PR = RF \cdot SSC$
Square root of Product of RF and SSC	S_PR	$S\_PR = \sqrt{RF \cdot SSC}$
Quotient of RF to SSC	QU	$QU = RF \cdot SSC^{-1}$
Square root of Quotient of RF to SSC	S_QU	$S\_QU = \sqrt{RF \cdot SSC^{-1}}$
Difference of RF to SSC	DI	$DI = RF - SSC$
Square root of Difference of RF to SSC	S_DI	$S\_DI = \sqrt{RF - SSC}$

Table S2 Resulting Staining conditions for each incubation time and dye using Visual Xsel parameter optimization tool. Abbreviations: c, fluorescence dye concentration; DMSO, Dimethyl sulfoxide; Isoprop, Isopropanol; t: Incubation time. Buffer types: phosphate buffer saline (PBS I) and phosphate buffer saline including 0.03 mg L^{-1} EDTA, and 0.08 mg L^{-1} Tris (PBS II); both adjusted to pH 7.5.

Dye	t [min]	C [µg mL^{-1}]	Buffer type	Biomass [µL]	DMSO [%]	Isoprop [%]
$BODIPY^{493/503}$	1	0.2	PBS II	500	10	9
	2	0.2	PBS II	500	10	10
	5	0.2	PBS II	500	9	10
	10	0.2	PBS II	500	5	10
LipidGreen2	1	2.0	PBS I	250	10	0
	2	1.7	PBS I	250	10	0
	5	1.0	PBS II	500	10	0
	10	1.1	PBS II	500	5	0
Nile red	1	1.4	PBS II	500	10	0
	2	1.3	PBS II	500	10	0
	5	1.0	PBS II	500	10	0
	10	1.0	PBS II	500	5	5
